# Nutrition literacy and its related demographic factors among workers of Taraz Steel company, Chaharmahal and Bakhtiari, Iran

**DOI:** 10.3389/fpubh.2022.911619

**Published:** 2022-08-12

**Authors:** Parastoo Yarmohammadi, Mohammad Ali Morowatisharifabad, Zohreh Rahaei, Sayyed Saeid Khayyatzadeh, Farzan Madadizadeh

**Affiliations:** ^1^Department of Health Education and Health Promotion, School of Public Health, Shahid Sadoughi University of Medical Sciences, Yazd, Iran; ^2^Department of Nutrition, School of Public Health, Shahid Sadoughi University of Medical Sciences, Yazd, Iran; ^3^Center for Healthcare Data Modeling, Departments of Biostatistics and Epidemiology, School of Public Health, Shahid Sadoughi University of Medical Sciences, Yazd, Iran

**Keywords:** nutrition literacy, socioeconomic status, workers, food, Iran

## Abstract

**Background:**

Nutrition is critical to prevent some chronic diseases. Nutrition literacy refers to ability to gain, understand and evaluate nutrition facts to choose appropriate foods. Nutrition literacy has recently drawn the attention of professionals with respect to health promotion. The purpose of this study was to investigate nutrition literacy and potentially related demographic factors among workers of a steel company in Chaharmahal and Bakhtiari province, southwest Iran.

**Methods:**

In this descriptive-analytical study in 141 workers of Taraz Steel company in 2021, participants were selected by convenience sampling and a self-report nutrition literacy scale nativized to Iranians was used to collect data. Data were analyzed by SPSS 22 using Mann-Whitney U test, Kruskal-Wallis test and Spearman's correlation coefficient.

**Results:**

About 75% of workers had adequate nutrition literacy and around 24% inadequate nutrition literacy. The highest percentage of mean score was obtained for determination of food groups (85.4%) and the lowest for calculation of food units (47%). The mean score of nutrition literacy was significantly higher in people with higher education (*P* = 0.020). Also, people with adequate monthly salary attained a higher mean score on determination of food groups (P = 0.021) and higher overall nutrition literacy (*P* = 0.003) compared to other people. No relationship was observed between nutrition literacy and body mass index as well.

**Conclusion:**

Most workers have adequate nutrition literacy but their scores on calculation of food units are relatively low. It is essential for policymakers to collect information on the level of nutrition literacy in different populations, especially Iranian workers, to reduce the prevalence of nutrition-related chronic diseases.

## Background

One per five deaths worldwide occurs due to unhealthy nutrition (1). Besides poor nutrition, obesity, and other nutrition-related conditions contribute significantly to poor health globally (2). In general, educational interventions contribute positively to increasing nutrition knowledge but do not necessarily result in enhanced dietary intake because of failure to link nutrition-related knowledge, skills and making critical decisions regarding dietary intake (3). Taken together, these concepts are called nutrition literacy and are critical to enhance the outcomes of relevant interventions. Nutrition literacy refers to ability to obtain, process, and understand nutrition information and skills needed to make appropriate nutrition decisions (4). Nutrition literacy refers to food-related knowledge and skills applied to select healthy diets including meal volume, knowledge on food labels, reliability of nutrition information, and rudiments of nutrition information (5, 6). Nutrition literacy assists in maintaining and improving health through selection of the right foods (7).

It has been found that individuals, subpopulations and communities with higher nutrition literacy are more able to understand the nutrition facts and consequently to follow a healthy diet (8). These people have higher knowledge about the relationship between poor diet and disease; therefore, nutrition literacy can help reduce disease burden and improve economic and health inequalities in poor communities (9). Low nutrition literacy may lead to adverse health impacts and increase in prevalence of chronic diseases (10, 11). As most chronic diseases can be prevented by proper diet, some research has shown a link between socioeconomic status (SES) and food quality, food selection, and nutrition literacy so that people of a low SES tend to consume lower-quality foods compared to those of a high SES (12).

Some studies have shown that nutrition literacy interventions may assist people of low SES in improving their nutrition (13, 14). In recent years, various tools have been developed and used to assess the level of nutrition literacy in different countries including Evaluation Instrument of Nutrition Literacy on Adults (EINLA) that is a commonly used and reliable research instrument to investigate nutrition literacy. The scale has been codified and validated for Turkish populations by Cesur et al. (15) and its nativized version to the Iranian population (16) was used in the present study.

Investigating the status of and gaining knowledge about nutritional literacy is critical to adopt effective educational interventions to improve the nutritional behavior and patterns of individuals, subpopulations and communities. A previous study in Iran showed that various inappropriate eating habits including high consumption of unhealthy foods, lack of eating breakfast, and low consumption of fruits, vegetables, dairy products, and whole grains are common inappropriate nutritional habits among Iranian adults (17).

According to the available evidence, few studies have so far been done to investigate nutritional literacy in the Iranian adults, especially the workers.

Because workers usually have a low SES and have comparably less time to think about their health and improve their nutritional health and nutritional behaviors due to busy schedules, appropriate educational interventions must be designed, evaluated and implemented to improve their nutritional literacy status through gathering of relevant information. The aim of this study was to investigate nutrition literacy and its relationship to demographic variables among workers of a steel company in Chaharmahal and Bakhtiari province, southwest Iran.

## Methods

A cross-sectional, descriptive study was conducted in workers of Taraz Steel company, Iran between January, 2021 and March, 2021. First, the study protocol was approved by the Vice Chancellor for Research and Technology of Shahid Sadoughi University of Medical Sciences (Ethics code: IR.SSU.SPH.REC.1399.187), and then the necessary coordinations were made to administer the questionnaires. To this end, the studied factory was visited in person. Due to the limited size of the study population, all workers (*n* = 141) were enrolled in the study by census sampling method. Inclusion criteria were being literate and volunteering to participate in the study, and the exclusion criteria were lack of complete filling out of the questionnaires and withdrawing from study.

After obtaining written informed consent to participate in the study from the workers, explaining the objectives of the research to them, and ensuring them of the confidentiality of their answers to the items, the researcher distributed the questionnaires among the workers and allowed them 10 min to fill out it. The questionnaire used was a self-report research instrument, and if there were any problems with understanding the items while filling out the questionnaire by the participants, the necessary explanations were provided by the researcher to them. All the questionnaires were given back and data drawn from 141 questionnaires were analyzed.

In this study, the EINLA was used to measure nutritional literacy (16). This questionnaire was developed and standardized in Turkey by Cesur et al. (15) and in Iran, Hemmati et al. nativized and used it to measure nutritional literacy of teachers. After translation-back translation, the reliability of the questionnaire was investigated by calculating its internal consistency (Cronbach's alpha coefficient = 0.73), showing the acceptable reliability of the tool for the Iranian population (16).

The questionnaire has two parts: (1) Demographic characteristics checklist (age, gender, education level, monthly salary, marital status, work experience, family dimension, working hours per day, shift work, medical history, smoking, physical activity, referring to physician, communicating with others and general health status) and (2) The main scale (consisting of 35 items) to measure nutrition literacy (16). The 35 items were divided into five sections: General nutrition information (10 items). This section addresses the respondent's nutritional knowledge (For example: Which are the best sources of healthy fat? a. Margarine, b. Animal fat, c. Corn oil, d. Olive oil).

Ability to read and understand nutrition (six items); in this section, first a short text was provided to the respondent and he/she was asked to answer the items related to the text. Understanding food groups (10 items); in this section, 10 images related to foods were provided to participants to assign them to the relevant food group. Determination of food groups (three items); in this section, the respondent answers the items related to the daily requirement of the body to each food unit (For example: milk and dairy products should be consumed daily. a. 1 unit, b. 2–3 units, c. 4 units, d. 5 units). Calculation of food units (six items); this section is aimed to evaluate ability to calculate body mass index (BMI) as well as ability to calculate the amount of calories and other information on a food label. Answers to the items are scored either 1 (correct) and 0 (incorrect/I don't know), with minimum and maximum attainable scores on the whole scale being 0 and 35, respectively. The cut-off point of the scale is 24; i.e., a score <24 indicates inadequate nutrition literacy and a score of 24 or higher does adequate nutrition literacy (18, 19).

Data were coded and then analyzed using SPSS version 22. First the normality of data distribution was first analyzed by the Kolmogorov-Smirnov test. Then Mann-Whitney U test was run to examine inter-group variance, Kruskal-Wallis test to do comparisons among three or more groups, and Spearman's correlation coefficient to examine the correlations between variables. *P* < 0.05 was considered significance level.

## Results

The mean age of our participants was 35.06 ± 5.85 years. Also their average work experience was 9.91 ± 6.09 years, their average family size 3.25 ± 1.57 individuals, and their average working h per day 9.91 ± 6.09 h. Other demographic information is shown in Table 1.

**Table 1:** Descriptive statistics of the participants socio-demographic and other variables.

**Variables**		***n***	**%**	**Variables**		***n***	**%**
Male	Gender	135	95.7	Smoking	Never	111	78.7
	Female	6	4.3		Infrequently	30	21.3
Education level	High school diploma	23	16.3	Physical activity	Daily	40	28.4
	Associate's degree	21	14.9		Several times a week	56	39.7
	Bachelor's degree	73	51.8		Once a month	26	18.4
	Master's degree and higher	24	17		Never	19	13.5
Monthly salary	Adequate	37	26.2	Physical activity	Daily	40	28.4
	Moderate	86	61		Several times a week	56	39.7
	Poor	18	12.8		Once a month	26	18.4
Marital status	Single	36	25.5		Never	19	13.5
	Married	105	74.5	Referring to physician	Less than three times a year	122	86.5
Work shift	Yes	74	52.5		More than three times a year	19	13.5
	No	67	47.5	Communicating with others	Frequently	73	51.8
History of disease	Yes	6	4.3		Moderately	61	43.3
	No	135	95.7		Infrequently	5	7
Physical activity	Daily	40	28.4	General health status	Very good	22	15.6
	Several times a week	56	39.7		Good	67.4	95
	Once a month	26	18.4		Moderate	24	17
	Never	19	13.5			

In general, the mean nutrition literacy of our participants was 27.10 ± 3.74 (range: 0–35). Around 75% (*n* = 106) of them had adequate nutrition literacy and about 25% (*n* = 35) inadequate nutrition literacy. The mean score of nutrition literacy was higher in individuals with postgraduate education than in other individuals (*P* = 0.02, Table 2).

**Table 2:** Comparison of average nutrition literacy and its dimensions in workers by demographic variables.

**Demographic**		**General**	**Ability to read**	**Determination**	**Calculation of**	**Ability to calculate food**	**Nutrition**
**variables**		**nutrition**	**and understand**	**of food**	**food units**	**units and read**	**literacy**
		**information**	**nutrition facts**	**groups**	**units**	**food labels**	**(total)**
**Education level**	High school diploma	7.73 ± 1.88	5.08 ± 0.79	8.39 ± 1.58	1.52 ± 0.66	3.56 ± 1.50	26.30 ± 4.40
	Associate's degree	8.04 ± 1.24	5.52 ± 0.81	8.61 ± 0.92	1.14 ± 0.65	4 ± 1.54	27.33 ± 1.87
	Bachelor's degree	7.83 ± 1.40	4.89 ± 1.13	8.50 ± 1	1.43 ± 0.76	4.06 ± 1.52	26.73 ± 3.80
	Master's degree and higher	8.45 ± 0.97	5.41 ± 0.71	8.75 ± 1.96	1.45 ± 0.88	4.70 ± 1.54	28.79 ± 3.77
*P*-value^a^		0.270	^*^0.013	0.060	0.272	0.055	^*^0.020
**Monthly income status**	Adequate	8.27 ± 1.38	5.35 ± 0.71	8.72 ± 1.59	1.48 ± 0.76	4.51 ± 1.53	28.35 ± 3.69
	Moderate	7.82 ± 1.31	5.02 ± 1.07	8.59 ± 0.59	1.36 ± 0.7	3.83 ± 1.54	26.63 ± 3.39
	Poor	7.94 ± 1.89	5 ± 1.13	7.94 ± 1.79	1.5 ± 0.98	4.38 ± 1.37	26.77 ± 4.94
*P*-value^a^		0.065	0.357	^*^0.021	0.648	0.055	^*^0.003
**Work shift**	Yes	7.85 ± 1.4	5.02 ± 1.03	8.55 ± 1.04	1.43 ± 0.74	3.85 ± 1.47	26.71 ± 3.48
	No	8.07 ± 1.43	5.19 ± 0.97	8.53 ± 1.53	1.38 ± 0.77	4.34 ± 1.59	27.53 ± 3.99
*P*-value^b^		0.194	0.261	0.157	0.539	^*^0.036	^*^0.043
**History of disease**	Yes	7.83 ± 1.47	4.66 ± 1.03	6.16 ± 3.54	1 ± 0.63	4.16 ± 1.83	23.83 ± 5.45
	No	7.96 ± 1.4	5.12 ± 1	8.65 ± 1	1.42 ± 0.75	4.08 ± 1.54	27.25 ± 3.61
*P*-value^b^		0.949	0.186	^*^0.003	0.174	0.822	0.111
**Mean** **±SD**		7.95 ± 1.41	5.10 ± 1	8.54 ± 1.29	1.41 ± 0.75	4.08 ± 1.54	27.1 ± 3.74
**Score range**		0–10	0–6	0–10	0–3	0–6	0-35
**Mean percentage of maximum attainable score (%)**		79.5	85	85.4	47	68	77.43

a *p–value was determined by Kruskal–Wallis test*.

b *p-value was determined by Mann-Whitney U test*.

* *Statistically significant (p value <0.05)*.

As well, individuals with a sufficient monthly salary had a higher mean score on determination of food groups (*P* = 0.021) and higher overall nutrition literacy (*P* = 0.003) compared to others. The mean score of ability to calculate food units and read food labels (*P* = 0.036) and total nutrition literacy (*P* = 0.043) was higher in workers without shift work than in those with shift work. The mean score on determination of food groups was higher in workers without a history of disease (*P* = 0.003). Other demographic variables (gender and marital status, smoking, level of physical activity, referring to physician, communicating with others and general health status) did not show a significant relationship to nutrition literacy and its dimensions (*P* > 0.05). The determination of food groups (85.4%) and the calculation of food units (47%) had the highest and lowest percentages of mean scores, respectively (Table 2).

Regarding the correlation between the five dimensions of nutrition literacy and total nutrition literacy, Age showed a direct, significant correlation with general nutrition information (*r* = 0.195). Also family size had an inverse, significant correlation with the ability to read and understand nutrition facts (*r* = −0.163) and determination of food groups (*r* = −0.184). Working h per day showed an inverse, significant correlation with determination of food groups (*r* = −0.165), ability to calculate food units and read food labels (*r* = −0.252), and total nutrition literacy (*r* = −0.186). There was no significant correlation between nutrition literacy scores and its dimensions and BMI (*P* > 0.05) (Table 3).

**Table 3:** Spearman's correlation coefficients between nutrition literacy scores and its dimensions with age, work experience, family size and working hours per day.

**Variable**	**General**	**Ability to read**	**Determination**	**Calculation of**	**Ability to calculate food**	**Nutrition**
	**nutrition**	**and understand**	**of food**	**food units**	**units and read**	**literacy**
	**information**	**nutrition facts**	**groups**	**units**	**food labels**	**(total)**
Age	0.195^*^	0.069	0.106	−0.079	−0.052	0.076
Body mass index	0.073	−0.016	−0.113	0.084	0.053	0.003
Work experience	0.039	0.063	0.051	−0.114	−0.029	0.007
Family size	−0.050	−0.163^*^	−0.184^*^	0.079	−0.006	−0.073
Working hours per day	−0.050	−0.091	−0.165^*^	0.120	−0.252^**^	−0.186^*^

* * Significant correlation at level 0.05*.

** *Significant correlation at level 0.01*.

## Discussion

To the best of our knowledge, the present study is first to investigate the nutrition literacy of Iranian workers. In this study, the participants' mean score of nutrition literacy was 27.10 ± 3.74 (maximum attainable score: 35) and most (77.43%) of them had satisfactory nutrition literacy. In agreement with the present study, the results of a study in Iran on 110 primary school teachers showed that 77.3% of them had adequate nutrition literacy (16). A study in Portuguese adults showed that almost half of participants had acceptable and high nutrition literacy (20). Besides that, 79.8% of people aged 18–64 (21) and 48% of adults in the lower Mississippi Delta, USA have been reported to have adequate nutrition literacy (22). This inconsistency in nutritional literacy levels obtained in different studies can be explained by the different characteristics of the study populations and the various nutritional literacy tools used.

Today, people can easily access information about nutrition and healthy eating habits through searching the Internet. Acceptable nutrition literacy represents the capability to understand nutrition information and food groups, read food labels, and identify appropriate food units, and ultimately to make informed decisions and/or support others in choosing the right food (22, 23). In the present study, the highest level of nutrition literacy was obtained for the determination of food groups, while the lowest level for the calculation of food units. In a study on seminarians in Iran, the highest score of nutrition literacy was obtained for the knowledge of food groups and the lowest score for the knowledge and skills of food units measurements (24). Other studies have shown that the minimum correct answers are related to items on the calculation of food units and the understanding and evaluation of food labels (16, 25).

High nutrition literacy is believed to help people choose healthier foods and consume fewer calories (19). Research in low-income adults has revealed that people with low nutrition literacy consume less fruits and vegetables than the recommended amounts. They are also less likely to appropriately read food labels (26). The findings of our study showed that there was a significant relationship between workers' education level and their nutrition literacy. Similarly, in other studies a direct correlation has been reported between the level of nutrition literacy and education level (16, 22). Two independent studies on American women and Taiwanese university students showed that people with higher education had better eating habits (27, 28). In other words, people with higher education are more able to gain and understand the nutritional information they need and use that information appropriately when making food-related decisions. Education is one of the most important determinants of health literacy and nutrition literacy. Therefore, planning for and implementing educational interventions for people with low nutrition literacy should be given priority to improve their nutrition literacy. The current study showed that people with low monthly salaries had lower nutrition literacy compared to other people. In this regard, one study in Greece showed that annual salary was inversely correlated with nutrition literacy (29). People with low income are more likely to adopt unhealthy eating habits and attitudes, and to have limited access to nutrition information and some foods, which can adversely affect their nutrition literacy. In the present study, no significant relationship was observed between nutrition literacy and BMI. Consistent with this finding, no significant relationship between BMI and nutrition literacy has been reported in similar studies (20, 21, 30). However, some other studies have shown a direct, significant correlation between nutrition literacy and BMI (8, 31). Because BMI is not affected only by the level of nutrition literacy, it seems that other factors and variables affecting BMI should also be studied in detail to arrive at more definitive conclusions. The administration of a self-report questionnaire and the relatively small sample size were two of the limitations of the present study.

## Conclusion

In this study, over half of the workers had adequate nutrition literacy and nutrition literacy was directly correlated with education level and monthly salary. It is essential to determine nutrition literacy in different demographic groups to identify people with low nutrition literacy so that effective educational interventions can be designed for them to improve their food-related behaviors and subsequently nutrition literacy. Improved nutrition literacy also assists in improving nutritional health in the long term. Taken together, policymakers in Iran should constantly take into account the nutrition literacy of different populations to improve their lifestyle and reduce the incidence of some chronic diseases in them.

## Data availability statement

The original contributions presented in the study are included in the article/supplementary material, further inquiries can be directed to the corresponding author/s.

## Ethics statement

The studies involving human participants were reviewed and approved by Ethics code (IR.SSU.SPH.REC.1399.187) by the Vice Chancellor for Research and Technology of Shahid Sadoughi University of Medical Sciences. The patients/participants provided their written informed consent to participate in this study.

## Author contributions

MM and PY study conception and design. FM acquisition and analysis of data. SK literature review. PY and ZR drafting and edition of manuscript and acquisition and analysis of data. All authors read and approved the final version of the manuscript.

## Conflict of interest

The authors declare that the research was conducted in the absence of any commercial or financial relationships that could be construed as a potential conflict of interest.

## Publisher's note

All claims expressed in this article are solely those of the authors and do not necessarily represent those of their affiliated organizations, or those of the publisher, the editors and the reviewers. Any product that may be evaluated in this article, or claim that may be made by its manufacturer, is not guaranteed or endorsed by the publisher.
